# Microbial Landscapes of the Gut–Biliary Axis: Implications for Benign and Malignant Biliary Tract Diseases

**DOI:** 10.3390/microorganisms13091980

**Published:** 2025-08-25

**Authors:** David Meacci, Angelo Bruni, Alice Cocquio, Giuseppe Dell’Anna, Francesco Vito Mandarino, Giovanni Marasco, Paolo Cecinato, Giovanni Barbara, Rocco Maurizio Zagari

**Affiliations:** 1 Department of Medical and Surgical Sciences, Universitaria di Bologna, 40126 Bologna, Italy; david.meacci@studio.unibo.it (D.M.); alice.cocquio@studio.unibo.it (A.C.); giovanni.marasco4@unibo.it (G.M.); giovanni.barbara@unibo.it (G.B.); roccomaurizio.zagari@unibo.it (R.M.Z.); 2 Gastroenterology Unit, IRCCS Azienda Ospedaliero-Universitaria di Bologna, 40138 Bologna, Italy; paolo.cecinato@aosp.bo.it; 3 Gastroenterology and Gastrointestinal Endoscopy Unit, IRCCS San Raffaele Hospital, 20132 Milan, Italy; dellanna.giuseppe@hsr.it (G.D.); mandarino.francesco@hsr.it (F.V.M.); 4 Gastroenterology and Gastrointestinal Endoscopy Unit, IRCCS Policlinico San Donato, 20097 Milan, Italy; 5 Faculty of Medicine and Surgery, Vita-Salute San Raffaele University, 20132 Milan, Italy; 6 Gastro-Esophageal Organic Diseases Unit, IRCCS Azienda Ospedaliero-Universitaria di Bologna, 40138 Bologna, Italy

**Keywords:** biliary microbiome, bile acids, oncobiome

## Abstract

Next-generation sequencing has overturned the dogma of biliary sterility, revealing low-biomass microbiota along the gut–biliary axis with metabolic and immunologic effects. This review synthesizes evidence on composition, function, and routes of colonization across benign and malignant disease. In cholelithiasis, *Proteobacteria*- and *Firmicutes*-rich consortia provide β-glucuronidase, phospholipase A_2_, and bile salt hydrolase, driving bile supersaturation, nucleation, and recurrence. In primary sclerosing cholangitis, primary biliary cholangitis, and autoimmune hepatitis, intestinal dysbiosis and disturbed bile acid pools modulate pattern recognition receptors and bile acid signaling (FXR, TGR5), promote Th17 skewing, and injure cholangiocytes; bile frequently shows *Enterococcus* expansion linked to taurolithocholic acid. Distinct oncobiomes characterize cholangiocarcinoma subtypes; colibactin-positive *Escherichia coli* and intratumoral Gammaproteobacteria contribute to DNA damage and chemoresistance. In hepatocellular carcinoma, intratumoral microbial signatures correlate with tumor biology and prognosis. We critically appraise key methodological constraints—sampling route and post-sphincterotomy contamination, antibiotic prophylaxis, low biomass, and heterogeneous analytical pipelines—and outline a translational agenda: validated microbial/metabolomic biomarkers from bile, tissue, and stent biofilms; targeted modulation with selective antibiotics, engineered probiotics, fecal microbiota transplantation, and bile acid receptor modulators. Standardized protocols and spatial, multi-omic prospective studies are required to enable risk stratification and microbiota-informed therapeutics.

## 1. Introduction

During the past decade, the exponential growth of microbiome research has forced the medical community to abandon the entrenched belief that many anatomical districts are intrinsically sterile. Culture-independent techniques, particularly high-throughput sequencing of 16S ribosomal RNA genes, have repeatedly disclosed resident microbial consortia in environments formerly judged incompatible with microbial life, including the biliary tract and the pancreatic ductal system [1,2]. The intestinal microbiota is currently acclaimed as the “last organ” of the human body because of its pervasive metabolic, endocrine, and immunological influence [3]. It regulates epithelial turnover, angiogenesis, entero-endocrine activity, neuro-humoral transmission, and bone remodeling; harvests energy from otherwise indigestible polysaccharides; biotransforms bile salts, drugs, and xenobiotics; neutralizes dietary toxins; and synthesizes vitamins, neurotransmitters, and myriad low-molecular-weight compounds whose biological targets are still being identified [4].

The gastrointestinal tract contains the most abundant and diverse fraction of the human microbiome. Under eubiotic conditions, commensals engage in continuous bidirectional communication with the epithelium and the underlying immune system, strengthen barrier integrity, out-compete opportunistic pathogens, and secrete short-chain fatty acids and other metabolites that nourish colonocytes and modulate systemic metabolism, thereby preserving mucosal homeostasis [5]. When community structure is deranged and host defenses falter, the epithelial barrier becomes leaky, bacterial translocation ensues, and microbe-associated molecular patterns activate Toll-like receptors (TLRs) on epithelial, immune, and stromal cells, driving chronic inflammation; genotoxin-producing bacteria such as colibactin-positive strains may even inflict direct DNA damage and chromosomal instability [4,6,7]. Given the intimate anatomical continuity of the duodenum, biliary tree, and pancreas, investigators have begun to explore whether the intestinal microbiota—or a discrete biliary or pancreatic microbiome—might confer comparable homeostatic functions on those organs [1,2].

Bile is an amphipathic fluid synthesized by hepatocytes to emulsify dietary lipids. Its principal organic constituents are bile acids (BAs), end products of cholesterol catabolism. Primary BAs, cholic (CA) and chenodeoxycholic acids (CDCA), are conjugated with glycine or taurine, which increases their solubility and acidity [1,2]. In the distal ileum and colon, members of the class Clostridia perform 7-α-dehydroxylation, generating the secondary BAs deoxycholic (DCA) and lithocholic acids (LA). Conjugated or unconjugated BAs can also be de-acylated by bile salt hydrolases (BSHs) expressed by Gram-positive genera such as lactobacilli, *Enterococcus, Bifidobacterium*, and *Clostridium* and by Gram-negative *Bacteroides*, thus reshaping the size and composition of the BA pool [8]. Free BAs are more hydrophobic and therefore more cytotoxic than their conjugated counterparts [9]. Alterations in the abundance of BSH- or bile acid-inducible enzyme-producing taxa dramatically reshape the enterohepatic BA pool and modify its receptor specificity. Quantitative or qualitative BAs disturbances have been implicated in autoimmune cholangiopathies, metabolic syndrome, inflammatory bowel disease, and *Clostridioides difficile* infection [10,11,12]. Secondary hydrophobic BAs also generate reactive oxygen and nitrogen species, thereby fostering carcinogenesis throughout the gastrointestinal tract [13].

Beyond their detergent action, BAs behave as versatile signaling molecules. Activation of the nuclear Farnesoid-X receptor or the membrane Takeda G-protein-coupled receptor 5 (TGR5) on monocytes and macrophages decreases phagocytic activity and inhibits NF-κB-dependent cytokine secretion, linking BA sensing to innate immunomodulation. Additionally, BAs at physiological levels block NLRP-3 inflammasome in macrophages, exerting anti-inflammatory properties, but when their systemic concentration increases, as in cholestasis, CDCA and DCA act as danger signals, activating pro-inflammatory pathways [9]. Therefore, BAs can function both as anti- and pro-inflammatory molecules depending on their systemic levels.

**Figure 1:**
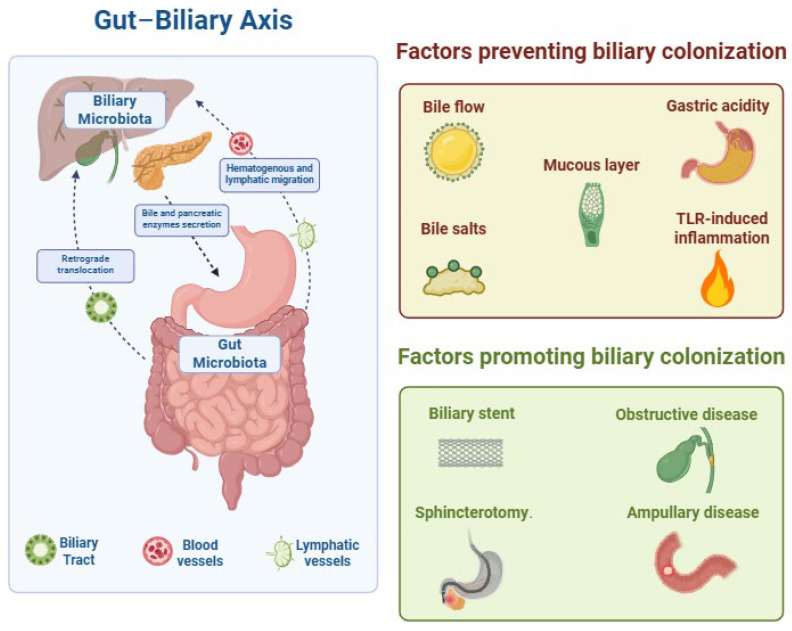
Gut–biliary axis: microbes can access the biliary tree via duodeno-biliary reflux and hematogenous or lymphatic translocation. Colonization is ordinarily restrained by antegrade bile flow, bactericidal bile salts, gastric acidity, the biliary mucous layer, and TLR-mediated epithelial immunity. Disruption of these defenses—by biliary stenting or endoscopic sphincterotomy, and in the context of biliary obstruction or ampullary disease—facilitates biliary colonization.

Several complementary barriers normally preserve sterility within the biliary tree. Continuous antegrade bile flow, assisted by gastric acidity and pancreatic enzymes, limits bacterial density in the proximal small bowel relative to the colon [14]. The amphipathic nature of BAs disrupts microbial membranes by solubilizing lipids and precipitating cell lysis [15]. The sphincter of Oddi provides an additional mechanical barricade; laxity of the sphincter, endoscopic sphincterotomy, biliary stenting, or obstructive processes such as choledocholithiasis and ampullary carcinoma markedly increase the risk of biliary contamination and infection (Figure 1) [16,17].

If microorganisms evade these first-line defenses, further safeguards come into play. Cholangiocytes exude a thick mucin layer that hampers adhesion, while tight junctions maintain epithelial cohesion and block paracellular leakage [14]. These cells also express a complete set of pattern recognition receptors (TLR1-TLR6 and TLR9) that initiate innate inflammatory responses, as illustrated in *Cryptosporidium parvum* infection [18]. Antimicrobial peptides, primarily β-defensin-1 and β-defensin-2, are constitutively produced along the intrahepatic tree, and the adaptive immune compartment delivers secretory IgA into bile, supplying an antibody shield against luminal antigens [15]. From the microbial perspective, survival in bile requires specific adaptations. Bacteria reinforce their envelopes by altering phospholipid composition; upregulate multidrug efflux pumps, porins, stress response proteins, and BSH activity; and deploy DNA repair pathways to mitigate BA-induced oxidative injury [8,19]. Gram-negative organisms are generally more resilient than Gram-positive ones: *Salmonella enterica*, *Escherichia coli*, and *Helicobacter pylori* withstand millimolar BA concentrations, whereas *Listeria monocytogenes*, *Enterococcus faecalis*, and several *Clostridium* species also show appreciable tolerance, albeit less pronounced [20,21,22,23,24,25]. Notably, bile tolerance is highly strain-specific, and in vitro resistance does not invariably translate into in vivo colonization.

Historically, these chemical and mechanical hurdles fostered the conviction that bile ducts were sterile [1]. Nonetheless, as early as 1967, Flemma et al. cultured bacteria from the bile of patients undergoing transhepatic cholangiography for obstruction yet lacking signs of cholangitis, coining the term “asymptomatic bactibilia” [26]. Modern sequencing studies have consistently confirmed that bile harbors microbiota, while simultaneously highlighting how little is known about the composition of a truly healthy biliary community, chiefly because bile is sampled only when disease is suspected [1,5]. In a porcine study, Jiménez et al. profiled bile, gallbladder mucus, and mucosa from healthy animals, and isolates were distributed almost equally among Firmicutes (34%), Actinobacteria (32%) and Proteobacteria (32%), whereas Bacteroidetes represented only 2%; *Streptococcus alactolyticus* accounted for more than 90% at the genus level [27]. Human data, though sparse, echo this complexity. Molinero et al. examined bile from 27 brain-dead liver donors and identified Actinobacteria, Firmicutes, and Bacteroidetes as the prevailing phyla in subjects without gallstones, with relative enrichment of Propionibacteriaceae and *Sphingomonas* compared with individuals harboring gallstones, suggesting that focal dysbiosis, rather than pure ascending infection, may underline certain biliary disorders [28].

Methodologically similar approaches have been extended to the pancreas. Culture and 16S rRNA sequencing of tissue obtained from organ donors, benign resections, or histologically normal margins of oncological specimens have detected microbial DNA in parenchyma previously presumed to be sterile [2]. Pushalkar et al. reported Firmicutes and Bacteroidetes as the dominant phyla, accompanied by *Brevibacterium*, *Chlamydiales*, *Mogibacterium*, *Oscillospira*, and members of the *Methylobacteriaceae* family [29], whereas Thomas et al. detected *Acinetobacter* and *Pseudomonas* as the most abundant genera [30,31].

The question of how microbes reach the pancreas remains incompletely resolved. The most intuitive explanation is retrograde migration through the pancreatic duct, which shares a common channel with the bile duct before emptying into the duodenum. In mouse models of pancreatic ductal adenocarcinoma whose resident microbiota had been ablated, oral gavage with *Bifidobacterium pseudolongum* resulted in sequential recolonization of the intestine and thereafter the pancreas, lending experimental support to a reflux pathway [29]. However, forced gavage is not a physiological event and cannot definitively establish causation. A second, non-exclusive mechanism involves hematogenous or lymphatic translocation from the gut. Bacteria routinely reach mesenteric lymph nodes, either directly or within migrating immune cells [32]. Diehl et al. showed that at a steady state, commensals inhibit trafficking of luminal bacteria to nodes; loss of Myd88 signaling or antibiotic-induced dysbiosis permits CX3CR1^hi mononuclear phagocytes to convey non-invasive bacteria to nodes via a CCR7-dependent pathway. Such lymphatic transport could in principle deliver microbes to the pancreas through established drainage channels, although definitive proof in humans is still lacking [33].

Collectively, current evidence indicates that neither bile ducts nor pancreatic parenchyma are sterile sanctuaries: each hosts dynamic microbial communities that interact with local metabolism and immunity. Deciphering the taxonomic composition, functional capacity, and temporal dynamics of these communities in health and disease may elucidate the pathogenesis of gallstone disease, cholangiopathies, pancreatitis, and pancreatic cancer and pave the way for microbiota-based diagnostics and therapies. A comprehensive understanding of host–microbe crosstalk along the gut–biliary axis therefore represents an urgent and promising frontier for translational research.

This narrative review aims to synthesize recent discoveries regarding the association between benign and malignant hepato-biliary conditions and the biliary and gut microbiota. Particular attention is paid to the potential causal role of microbiota in these diseases’ pathogenesis. Moreover, this article explores microbiota as a possible tool for diagnosis and prognosis, as well as a potential therapeutic target.

## 2. The Microbiome and Primary Sclerosing Cholangitis

Primary sclerosing cholangitis (PSC) is a rare chronic cholestatic liver disease characterized by chronic fibro-inflammatory damage of intra- and extrahepatic biliary ducts with invariable progression to end-stage cirrhosis. It is strongly associated with a unique phenotype of inflammatory bowel disease (IBD): pancolitis with right-sided predominance [34]. Etiopathogenesis is still not completely understood. Genetic studies support the hypothesis that PSC is an autoimmune disorder, but its male preponderance and unsatisfactory response to immunosuppressive therapy challenge this thesis, suggesting involvement of alternative and/or additional pathogenetic mechanisms [35,36].

Growing evidence indicates that dysbiosis of the gut microbiota plays a key role in PSC and other immune-mediated liver diseases, not only through translocation of bacteria and their products across a leaky gut barrier to the liver, where they trigger inflammatory responses, but also through induction of intestinal immune imbalance and shifting of the BA pool toward more cytotoxic species (secondary hydrophobic BA) [12,37]. Several next-generation sequencing (NGS) studies [38,39,40,41,42,43,44,45,46,47,48] performed on stool and mucosa samplings in PSC patients have shown that the alpha-diversity of the intestinal microbiota is significantly reduced and its composition differs from that seen in healthy volunteers, IBD alone, and other chronic liver diseases [5,49], although the difference between PSC only and PSC-IBD appeared to be marginal, indicating that liver pathology is the principal corollary of microbial dysbiosis (Table 1) [50].

A fairly consistent observation, despite the overall heterogeneity of results, was the abundance of *Veillonella*, reported to be 4.8-fold higher in PSC patients compared to healthy controls (*p* < 0.0001) in the 2017 study by Kummen et al. [40], even though this finding may not be PSC-specific but potentially a biomarker of advanced liver disease [51,52,53]. Smaller increases were also noted in *Enterococcus*, *Streptococcus*, lactobacilli levels, and members of the Proteobacteria phylum, such as *Escherichia coli*, alongside a notable depletion of short chain fatty acid (SCFA)-producing Firmicutes, such as *Faecalibacterium*, *Roseburia*, and *Coprococcus*. Collectively, these changes result in a depletion of microbial species with anti-inflammatory and immunomodulating effects and in an abundance of taxa involved in the dehydroxylation of primary bile acids and synthesis of amine oxidases, processes that contribute to inflammation and fibrosis [37,49,54,55]. As for the mycobiome, there is emerging evidence of fungal gut dysbiosis in PSC: Lemoinne et al. demonstrated an increase in the proportion of *Exophiala* and a decrease in *Saccharomyces cerevisiae*, known to have anti-inflammatory properties [56], while Bang and colleagues reported an increase in *Candida* species [57], which seems to be associated with more severe cholangitis [58].

Currently, the only life-saving treatment for PSC patients is orthotopic liver transplantation. However, microbiota modulation therapies, such as antibiotic administration of Vancomycin [37] and fecal microbiota transplantation [59], are emerging therapeutic options, whose beneficial effects are likely mediated by a shift in the gut microbiome, with favorable effects on BA metabolism [37,49,51,60]. Yet, long-term benefits remain unproven.

While the role of colonic microbiota in the pathophysiology of PSC is well-established, a novel promising area of research is represented by the biliary microbiome. To date, limited evidence exists on its composition and pathogenetic relevance in PSC, which is not surprising considering that biliary ducts were long regarded as a sterile compartment, and the microbial profile of healthy bile remains largely unknown. In earlier culture- and PCR-based studies [61,62,63], the most frequently isolated bacteria were *Streptococcus*, *Enterococcus*, and *Staphylococcus*. The latest NGS studies [36,64,65,66] show conflicting evidence.

In a 2017 pilot study, Pereira et al. [64] found no significant differences in bacterial communities in controls and early-stage PSC patients (the most common genera were *Prevotella*, *Streptococcus*, *Veillonella*, *Fusobacterium*, and *Haemophilus*). However, the groups with advanced-stage disease and dysplasia/neoplasia showed significant enrichment in several microbial taxa, most notably *Streptococcus*, compared to the early-stage group. These findings suggest that *Streptococcus* might play a role in promoting disease progression rather than contributing to the initial onset of PSC.

In contrast, in the 2020 study by Liwinski et al. [36], performed on a cohort of 43 PSC patients without bile duct dysplasia or CCA, the biliary microbiota displayed ecological alterations compared to healthy controls, such as reduced biodiversity and expansion of pathobionts. Moreover, *Streptococcus* was the predominant genus in both the PSC and control cohorts, but the former showed a significant increase in *Proteobacteria* (25% vs. 12%) and, at the genus level, *Enterococcus* (especially *E. faecalis*), *Staphylococcus*, and *Neisseria.* Bile cultures showed a trend towards higher levels of known pathogenic bacteria (*Enterococcus* spp., *Klebsiella* spp., *Enterobacter cloacae*, *Citrobacter freundii*, or *Staphylococcus* spp). Consistent findings were also reported in a smaller study by Tyc et al. [65], with *Streptococcus* as the predominant genus in both the PSC and control cohorts. Another notable finding that emerged from this study was the strong correlation between *Enterococcus* abundance and elevated levels of taurolithocholic acid (TLCA), a cytotoxic and potentially carcinogenic bile acid, suggesting that a dysbiotic bile microbiome—particularly *E. faecalis*—may promote epithelial damage and immune activation via TLCA production, along with metalloproteinases release [67] and induction of Th17 response [62]. In support of this observation, an association between biliary colonization by *Enterococci* and disease progression was demonstrated in a recent study by Zigmond et al. [68]. Yet, a recent study by Miyabe et al. [66] found no statistically significant differences between PSC patients’ and controls’ bile samples but identified an association between increased species richness—particularly of *Fusobacteria*—and the duration of PSC and CCA, raising the possibility of an inflammation-driven carcinogenic role of microbiota in CCA.

This heterogeneity across studies may be attributed to multiple factors, including variations in experimental protocols, selection bias due to the inability to ethically perform ERC in healthy controls, difficulty in obtaining bile samples in a sterile way [36], administration of perioperative antibiotic prophylaxis, and the high rate of false positive signals resulting from contamination and inefficiencies in the analyses of low-biomass microbial communities, such as those found in the biliary tract [69].

**Table 1:** Biliary microbiome in primary sclerosing cholangitis.

Reference (Year)	Cohort/Sample Type	Sequencing or Culture Technique	Biodiversity vs. Controls	Dominant Genera in Controls	Alterations in PSC	Clinical Correlations
Pereira et al., 2017 [64]	10 early-stage PSC vs. 9 gallstone controls; ERC bile	16S rRNA NGS	No significant difference	*Prevotella*, *Streptococcus*, *Veillonella*, *Fusobacterium*, *Haemophilus*	Increased concentration of *Streptococcus* in dysplasia/CCA	Early disease shows near-physiological biliary community
Liwinski et al., 2020 [36]	43 PSC (no dysplasia or CCA) vs. 34 controls; ERC bile	16S rRNA NGS + quantitative culture	Reduced richness; higher intra-patient variability	*Streptococcus *predominant	Proteobacteria 25% (vs. 12%); *Enterococcus* *faecalis*, *Staphylococcus*, *Neisseria* ↑	*Enterococcus* burden strongly correlates with TLCA levels, metalloproteinase activity, and accelerated fibrosis
Tyc et al., 2021 [65]	20 PSC; ERC bile	16S rRNA NGS	Markedly reduced		Expansion of *Proteobacteria* pathobionts mirrored Liwinski’s results	Supports link between Enterococcaceae dominance, Th17 polarization, and duct injury
Miyabe et al., 2024 [66]	32 PSC vs. 20 choledocholithiasis controls; ERC bile	16S rRNA NGS	Similar overall diversity	*Streptococcus *core genus	Species richness, especially *Fusobacteria*, rises with PSC duration and CCA	Suggests inflammation- driven microbiota maturation contributes to oncogenesis
Early Culture or PCR studies 1998–2010 [61,62,63]	76 PSC; bile or brushings	Aerobic or anaerobic culture, species- specific PCR			Frequent isolation of *Streptococcus*, *Enterococcus*, *and Staphylococcus*	First evidence that bile is not sterile and harbors opportunistic Gram-positive cocci

Abbreviations: CCA = cholangiocarcinoma; ERC = endoscopic retrograde cholangiography; NGS = next-generation sequencing; PSC = primary sclerosing cholangitis.

## 3. Primary Biliary Cholangitis

Primary biliary cholangitis (PBC) is a chronic inflammatory autoimmune cholestatic liver disease, characterized by lymphocytic inflammation and fibrotic destruction of small to medium-size intrahepatic bile ducts, resulting, if untreated or in case of poor response to therapy, in cholangitis, liver fibrosis, and eventually cirrhosis [70,71]. Although not yet fully elucidated, pathogenesis is multifactorial and arises from the complex interplay between immune and cholestatic pathways, shaped by both genetic susceptibility and environmental factors [71,72]. Ursodeoxycholic acid (UDCA), a synthetic bile acid, represents the gold-standard therapy.

As seen for PSC, growing attention has been directed toward the potential pathogenetic role of gut dysbiosis in PBC. Several NGS studies have investigated gut microbial profile in both treatment-naïve and UDCA-treated patients [72,73,74,75,76]. Tang et al. [74] found a reduction in species richness in untreated PBC patients, as well as a significant shift in overall microbial composition towards pro-inflammatory and potentially pathogenic taxa, with a decrease in *Faecalibacterium* and Oscillospirales, in line with findings reported by Zhou et al. [72] and Chen et al. [76], and an increase in *Veillonella*, *Streptococcus*, *Haemophilus*, *Clostridium*, *Klebsiella*, *Pseudomonas*, and certain Lactobacillales genera. Gut dysbiosis was partially restored after 6 months of treatment with UDCA, with an increase in some of the previously depleted genera. Of note, *Faecalibacterium* was particularly depleted in gp210-positive patients, a subgroup typically associated with more severe disease. This finding was consistent with the study by Furukawa et al. [75], which reported lower *Faecalibacterium* abundance among UDCA non-responders. This subgroup also exhibited lower levels of the Oscillospiraceae family compared to gp210-negative patients, suggesting a potential prognostic value for these two microbial biomarkers [72]. Furthermore, Zhou et al. identified *Serratia*, Oscillospirales, Ruminococcaceae, *Faecalibacterium*, Sutterellaceae, and *Coprococcus* as optimal microbial biomarkers for distinguishing PBC patients from healthy controls, achieving an area under the curve (AUC) of 0.824 [72].

A very recent study by Han et al. [77] provided a more detailed characterization of both the microbial and metabolic profiles of patients with poor biochemical response to UDCA, confirming a marked reduction in beneficial taxa—particularly *Gemmiger*, *Faecalibacterium*, and *Blautia*—alongside an increase in potentially pathogenic genera, especially *Ruminococcus *and *Clostridium*, on stool samples. These microbial alterations were accompanied by a disrupted bile acid composition, notably a reduced secondary/primary BA ratio, which may contribute to disease progression through the loss of the immunoregulatory functions typically exerted by secondary BAs [76,77].

To the best of our knowledge, only two early works [61,78] have investigated the biliary microbiota in PBC, and the available evidence remains limited and inconclusive, likely due to the technical challenges associated with endoscopic access and bile sample collection. In their study, Hiramatsu et al. analyzed gallbladder bile samples from 15 patients with primary biliary cirrhosis undergoing liver transplantation and detected bacterial DNA in 66% of samples, compared to only 8% in healthy controls (*p* < 0.01). The most abundant taxa were Gram-positive cocci, such as *Staphylococcus aureus* and *Enterococcus faecium*, suggesting a potential role of these bacteria in the etiopathogenesis of the disease, although the authors acknowledged that their presence could reflect secondary colonization due to impaired bile flow in advanced stages of the disease [78]. In contrast to this, Olsson et al. found no viable bacteria in bile ducts of explanted livers from PBC patients, as opposed to PSC samples, which frequently contained cultivable pathogens [61].

In conclusion, accumulating evidence supports the presence of gut dysbiosis in PBC; however, whether this represents a primary pathogenetic factor or instead a consequence of bile acid alteration is still to be clarified, given the bidirectional relationship between gut microbiome and BAs [5,12]. In addition, further investigations on the PBC biliary microbiome are warranted, as they may provide insights that complement and expand upon those obtained from fecal microbiota profiling.

## 4. The Microbiome and Autoimmune Hepatitis

Autoimmune hepatitis is a chronic progressive immune-mediated liver disease characterized by a female predominance, hypergammaglobulinemia, circulating autoantibodies, and a generally favorable response to immunosuppressive treatment, although the high recurrence rate often makes long-term immunosuppression necessary [79]. As with most autoimmune diseases, the precise mechanisms underlying the breakdown of immune tolerance are not fully elucidated yet, although genetic predisposition, environmental factors (e.g., antibiotics and molecular mimicry), and impaired control of regulatory T-cells are thought to play a central role [79,80].

Recent scientific and technological advances, especially in the genome-sequencing field, have led to an increasing recognition of the contribution of gut dysbiosis to the onset and progression of AIH. Several studies have evaluated the composition of gut microbiota in AIH patients [81,82,83,84,85], consistently reporting reduced bacterial diversity and disease-specific taxonomic alterations compared to healthy controls and PBC patients, which were partially restored after azathioprine treatment [83]. Common findings across studies were enrichment in *Veillonella*, *Klebsiella*, *Streptococcus*, *Haemophilus*, and lactobacilli and depletion in *Ruminococcaceae*, *Faecalibacterium*, and *Bifidobacterium*. Wei et al. found that the combination of four genera (*Veillonella*, Clostridiales, *Oscillospira*, and lactobacilli) could discriminate AIH from controls, with an AUC of 0.78 [82]. These changes in microbial composition were functionally mirrored by an over-representation of LPS biosynthesis, decreased production of SCFA, and alteration in amino acid metabolism.

In addition, gut microbial diversity appears to have a prognostic significance; according to Liwinski et al., a lack of *Bifidobacterium* has been associated with increased disease activity and failure to achieve remission [83], whereas overabundance of *V. dispar*, the most strongly disease-associated taxa, has shown a positive correlation with elevated levels of aspartate aminotransferase (AST) and liver inflammation [82].

Notably, there is a key contradiction between the studies by Liwinski et al. [83] and Chen et al. [76], reporting a classical dysbiotic profile with a lack of the SCFA-producing genus *Faecalibacterium*, and those by Lou et al. [81] and Elsherbiny et al. [84], which instead observed an over-representation of *Faecalibacterium* in AIH patients, potentially reflecting a compensatory mechanism.

In conclusion, an accumulating body of evidence demonstrates the pivotal role of intestinal microbiota in the pathogenesis of AIH; at the same time, gut microbiota emerged as a promising non-invasive diagnostic biomarker [80], although the disparate and sometimes contradictory evidence warrants further larger studies to validate disease-specific microbial signatures [50,80].

To date, no published studies have systematically investigated the biliary microbiota in AIH. Possible reasons for this gap are the limited access to biliary samples, as AIH patients are rarely subjected to ERCP, along with the fact that AIH is primarily a hepatocellular disease, so bile duct involvement is secondary and minimal.

## 5. The Oncobiome and Hepato-Biliary Malignancies

The human microbiome plays a pivotal role in maintaining health and contributing to disease pathogenesis. In recent years, microbiota has been increasingly recognized as a potential factor involved in the development and progression of various neoplastic conditions. This has led to the emergence of the term *oncobiome*, referring to the collective community of bacteria, viruses, and fungi that may exert either promotive or suppressive effects on tumor initiation, progression, metastasis, and therapeutic response [86]. Several mechanisms have been proposed through which microbiota may influence carcinogenesis, including modulation of tumor-related signaling pathways, alteration of the tumor microenvironment (TME), induction of DNA damage, and promotion of metastatic spread [87,88,89,90]. For instance, Chen et al. have demonstrated that *Fusobacterium nucleatum* is capable of reducing the expression of several molecules involved in epigenetic modulation, such as m^6^A and METTL3, thereby stimulating the YAP/KIF26B pathway and conferring to the colorectal cancer cells cultured with this bacterium, a benefit in terms of aggressiveness and metastatic capacity [88]. Moreover, *F. nucleatum* can modulate TME through the expression of Fap2 protein, which interacts with TIGIT receptor on NK cells and intratumoral lymphocytes, favoring the immunological escape of neoplastic cells [89].

Specifically, dysbiosis of the microbiota has been implicated in malignancies arising from the biliary system and hepatic parenchyma, including hepatocellular carcinoma, cholangiocarcinoma, and gallbladder cancer [91,92,93].

### 5.1. The Microbiome and Hepatocellular Carcinoma

Hepatocellular carcinoma (HCC) is the most common primary liver malignancy and represents the second most frequent hepatic neoplasm after metastatic liver cancer [94,95]. Globally, HCC ranks as the sixth most prevalent cancer and the third leading cause of cancer-related mortality [96]. Typically, HCC arises in the context of liver cirrhosis, making any etiology of cirrhosis a significant risk factor for its development [97].

To the best of our knowledge, no studies to date have specifically investigated the role of the biliary microbiota in HCC pathogenesis. However, emerging evidence suggests that the intratumoral microbiome—believed to originate from the gut, oral cavity, and biliary tract—plays a crucial role in modulating tumor progression, therapeutic responsiveness, and long-term prognosis in HCC patients [98].

The first investigation into HCC-associated intratumoral microbiota was conducted by Komiyama et al., who compared microbial communities in tumor tissues with those in adjacent nontumorous liver tissues. Their findings demonstrated a significantly higher microbial load within tumor samples [99]. Neoplastic tissues may offer a permissive microenvironment for microbial colonization and proliferation due to the presence of neoangiogenesis, hypoxic niches favoring anaerobic and facultative anaerobic bacteria, and necrotic areas conducive to microbial growth [100]. The predominant bacterial phyla identified in HCC tissues include Firmicutes, Bacteroidetes, and Proteobacteria [99]. Compared to adjacent non-neoplastic liver tissue, HCC samples exhibit an increased relative abundance of Enterobacteriaceae, *Clostridium*, *Fusobacterium*, and *Neisseria* genera, along with a marked reduction in the Pseudomonadaceae family [101]. Moreover, the HCC-associated microbiome is characterized by both increased microbial abundance and reduced species diversity [102]. Several specific microorganisms have been implicated in hepatocarcinogenesis. For instance, *E. coli* produces cytotoxic compounds such as cytolethal distending toxin (CDT), which may promote chronic inflammation and induce DNA damage, thereby facilitating tumor progression [103]. *F. nucleatum* also possesses oncogenic properties, including the activation of the ERK and STAT3 signaling pathways and the modulation of the TME toward an immunosuppressive phenotype [104]. Additionally, dysbiosis in HCC has been linked to aberrant activation of hepatic stellate cells through mechanisms associated with cellular senescence, further contributing to carcinogenesis [105]. Conversely, certain bacteria commonly depleted in HCC tissues, such as *Pseudomonas* spp., appear to exert antitumoral effects. *Pseudomonas aeruginosa*, for example, produces tumoricidal agents like azurin and exotoxins that promote apoptosis in cancer cells. Notably, HCC patients with higher intratumoral levels of *Pseudomonas* have demonstrated improved overall survival compared to those with lower levels [106].

HCCs arising from different clinical backgrounds exhibit distinct intratumoral microbiota profiles. For instance, *Ruminococcus gnavus* has been identified as a signature determinant in HCC associated with hepatitis B or C virus (HBV/HCV)-related liver disease [99]. In contrast, *Staphylococcus* and *Caulobacter* genera appear to be selectively enriched in HBV-negative HCC cases [107].

Moreover, distinct patterns of intratumoral microbiota have been associated with varying prognoses and clinical outcomes in HCC. Li et al. analyzed liver samples from 29 patients with HBV-related HCC, evaluating paired tumor and adjacent nontumorous tissues. Based on intratumoral microbiota composition, two distinct HCC subtypes were identified: a *viral-dominant* and a *bacteria-dominant* subtype. The bacteria-dominant subtype was significantly associated with larger tumor diameter (*p* = 0.041), elevated plasma D-dimer levels (*p* = 0.047), a lower relative proportion of macrophages in the tumor microenvironment (TME) (*p* = 0.015), and increased capsular invasion (*p* = 0.027). Importantly, this subtype was also linked to a significantly shorter disease-free survival following surgical resection compared to the viral-dominant subtype [102]. Similarly, Sun et al. investigated 91 HCC patients undergoing hepatectomy and identified two distinct microbial-based tumor subtypes, referred to as *hepatotype A* and *hepatotype B*. Hepatotype B was characterized by higher alpha-diversity, greater abundance of specific bacterial families such as *Methylobacterium* and *Akkermansia*, and reduced presence of *Proteobacteria* and *Actinobacteria* compared to hepatotype A. Notably, hepatotype A was associated with a poorer prognosis, including significantly reduced overall survival (*p* = 0.006) and shorter recurrence-free survival [108]. In a recent study, Jiang et al. evaluated the prognostic role of specific bacterial taxa in 172 patients with HCC who underwent surgical resection. Patients were stratified into two groups based on overall survival at 36 months: short overall survival (SOS) and long overall survival (LOS). The SOS group exhibited a lower abundance of *Brachybacterium* (*p* = 0.00392) and *Rothia* (*p* = 0.0740) and a higher abundance of *Intestinomonas* (*p* = 0.00925). These three taxa were identified as key microbial discriminants between the two prognostic groups [109].

### 5.2. The Microbiome and Cholangiocarcinoma

Cholangiocarcinoma (CCA) is a malignant neoplasm arising from the epithelial lining of the intrahepatic or extrahepatic bile ducts. Based on its anatomical site of origin, CCA is classified into three main subtypes: intrahepatic (iCCA), perihilar (pCCA), and distal (dCCA). It represents the second most common primary liver malignancy and is associated with a poor prognosis, with a 5-year overall survival rate of less than 10% [110,111]. Risk factors for CCA development include genetic predisposition, chronic biliary inflammation (e.g., primary sclerosing cholangitis), environmental exposures, and parasitic infections such as *Opisthorchis viverrini* and *Clonorchis sinensis* [112,113,114].

The growing recognition of the bile duct as a potential niche for microbial colonization has led to increasing interest in the role of the biliary microbiota in CCA tumorigenesis (Table 2). Ito et al. investigated the biliary microbiota of patients with biliary malignancies and compared it with that of individuals with benign biliary diseases. Their findings revealed a significant enrichment of the Enterobacteriaceae family in bile samples from patients with malignant conditions. Furthermore, metagenomic analysis identified the presence of colibactin-producing *Escherichia coli* in the malignant group [115]. Colibactin is a genotoxic peptide capable of inducing DNA damage and potentially promoting carcinogenesis [116]. Additional evidence supporting the increased abundance of *Enterobacteriaceae* in patients with dCCA comes from a study by Chen et al., who compared the biliary microbiota between dCCA patients and those with choledocholithiasis. In the dCCA group, bile samples showed not only a higher abundance of Enterobacteriaceae but also greater microbial diversity and elevated levels of *Staphylococcus*, *Klebsiella*, *Faecalibacterium*, and *Corynebacterium* compared to the benign disease group [7]. Multiple studies have consistently identified Enterobacteriaceae as one of the most prevalent bacterial families in CCA patients, and some genera, such as *Enterobacter* and *Pseudomonas*, have been proposed as potential microbial biomarkers for CCA [93]. In this context, Di Carlo et al. reported that, among patients with extra-pancreatic biliary cancers, CCA was associated with increased abundance of *Alcaligenes faecalis* and reduced levels of *Acinetobacter* spp. compared to patients with pancreatic neoplasms. These microbial differences may offer a useful tool for differential diagnosis between these malignancies [117]. Further support for the diagnostic utility of biliary microbiota comes from a study by Li et al., which analyzed and compared the microbial profiles in pCCA, dCCA, and pancreatic cancer. Their findings revealed that dCCA samples were enriched in *Streptococcus*, *Prevotella*, and *Halomonas*, while pCCA samples demonstrated higher abundance of *Pseudomonas*, *Sphingomonas*, and *Halomonas*. These distinct microbial signatures may contribute to more accurate classification and differentiation of biliary and pancreatic malignancies [118].

Additional evidence suggests a potential role for *Helicobacter pylori* in the pathogenesis of CCA. Avilés-Jiménez et al. analyzed samples collected via endoscopic retrograde cholangiopancreatography (ERCP) brushings from 100 patients with CCA and a comparable number of controls with benign biliary diseases. Patients with CCA exhibited a significantly higher relative abundance of several microbial taxa, including *Helicobacter*, *Campylobacter*, *Prevotella*, *Fusobacterium*, and members of the Methylophilaceae family. Notably, the study also investigated the presence of two major *H. pylori* virulence factors—*cagA* and *vacA*—in these samples. Both virulence genes were detected in approximately 50% of CCA patients, suggesting a potential link between *H. pylori* infection and carcinogenesis in the biliary tract. Interestingly, *cagA* and *vacA* were also identified in 42% and 25% of patients with benign biliary diseases, respectively—conditions which are themselves recognized as risk factors for CCA development. These findings raise the possibility that chronic colonization by *H. pylori*, particularly strains harboring these virulence factors, may contribute to the progression from benign biliary pathology to malignancy [119].

In conclusion, the intratumoral microbiota of CCA appears to influence both tumor progression and therapeutic response. CCA tumors resistant to standard chemotherapeutic agents such as gemcitabine and cisplatin have been shown to contain a higher abundance of Gammaproteobacteria compared to treatment-sensitive tumors. This bacterial group includes species capable of producing cytidine deaminase, an enzyme that can inactivate nucleoside analog chemotherapeutics like gemcitabine. The presence of such bacteria may contribute to a tumor microenvironment that fosters chemoresistance, ultimately reducing the efficacy of standard treatments and adversely affecting patient outcomes [120].

### 5.3. The Microbiome and Gallbladder Cancer

Gallbladder carcinoma (GC) is a rare malignancy that typically originates from the gland’s papillary epithelial cells, which are involved in bile motility [121]. Although uncommon, GC is associated with a poor prognosis, primarily due to its tendency to be diagnosed at an advanced or metastatic stage. The median survival time following diagnosis ranges from 6 to 12 months [122]. Known risk factors for GC include gallstone disease, advanced age, obesity, genetic predisposition, and certain infections or bacterial colonization of the bile. These observations support a potential role for the biliary microbiota in the pathogenesis of gallbladder carcinoma [121].

Various studies have reported an association between *Helicobacter bilis* colonization and biliary tract diseases, particularly gallbladder and bile duct cancers. *H. bilis* has been shown to activate multiple oncogenic signaling pathways, including NF-κB and VEGF, which may contribute to GC development [123,124]. Another notable microbiological risk factor for GC is chronic infection and carriage of *Salmonella typhi*. Typhoid carriers have an estimated 6% lifetime risk of developing gallbladder cancer, a risk believed to be driven by persistent bacterial colonization, which induces DNA damage and alters bile composition—both of which promote carcinogenesis [125,126,127].

In recent years, a growing body of evidence has supported the involvement of the biliary microbiota in the pathogenesis of GC, highlighting the presence of a distinct microbial signature in affected patients. Tsuchiya et al. reported a significantly higher prevalence of bacterial colonization in the bile of GC patients compared to individuals with gallstones (42.9% vs. 13.3%). Microbial profiling revealed that bile samples from GC patients were enriched in *F. nucleatum*, *Enterobacter* spp., and *Escherichia coli*. In contrast, patients with cholelithiasis showed a dominance of *Enterococcus gallinarum*, *Salmonella* spp., and *Escherichia coli* [128].

Another study demonstrated a progressive increase in microbial abundance in bile samples along the spectrum from healthy individuals to patients with chronic cholecystitis and, ultimately, GC. Patients with chronic cholecystitis exhibited the presence of bacterial genera such as *Enterococcus*, *Citrobacter*, and *Klebsiella* in their bile. Notably, *Klebsiella* species were found in significantly higher abundance in the bile of GC patients, alongside other members of the Enterobacteriaceae and Streptococcaceae families. This stepwise enrichment of *Klebsiella* from a normal to an inflammatory and finally to a malignant state supports the hypothesis that this genus may play a contributory—if not causative—role in gallbladder carcinogenesis [129].

Finally, a study by Song et al. involving patients with chronic cholecystitis and GC demonstrated that the core bacterial components of bile were largely similar between the two groups. However, GC samples exhibited reduced microbial species richness, suggesting a loss of diversity in the malignant state. Moreover, notable differences in microbial composition were observed: *Fusobacterium mortiferum*, *Peptostreptococcus stomatis*, and *E. faecium* were more abundant in the GC group. These bacterial species have previously been implicated in other malignancies—*E. faecium*, for instance, has been associated with enhanced responsiveness to immunotherapy in metastatic melanoma, while *P. stomatis* plays a significant role in the pathogenesis of gastric and colorectal cancers. Their enrichment in GBC may therefore reflect a broader role in cancer progression across multiple organ systems [130].

## 6. Conclusions

The evidence amassed over the past decade converges on a fundamental paradigm shift: neither the biliary tract nor the pancreatic parenchyma is intrinsically sterile. Instead, each hosts low-biomass but metabolically active microbial communities that engage in continuous crosstalk with epithelial, immune and stromal compartments, modulate bile acid pools, and, when dysbiotic, precipitate chronic inflammation, fibrosis, and tumorigenesis. Discrete taxonomic signatures—such as the Proteobacteria- and *Veillonella*-enriched pattern that typifies primary sclerosing cholangitis, or the Enterobacteriaceae-dominated profile of cholangiocarcinoma—have begun to delineate disease-specific onco-, fibro-, and immunobiological pathways. At the same time, functional read-outs illustrate how shifts in bile salt hydrolase, 7-α-dehydroxylase, and genotoxin production re-shape the enterohepatic “metabotype,” forging mechanistic links to epithelial barrier failure, Th17 polarization, DNA damage, and chemoresistance. The clinical corollary is clear: rigorous, spatially resolved mapping of the gut–biliary axis is now indispensable for refining risk stratification, biomarker discovery, and microbiota-targeted therapeutics. In this context, the latest multidimensional appraisals of therapeutic endoscopic ultrasound underscore its dual diagnostic and interventional potential, not only for restoring luminal patency but also for procuring uncontaminated bile and tissue samples along otherwise inaccessible segments of the ductal tree [131,132].

However, numerous methodological limitations should be considered when interpreting current findings. First of all, the heterogeneity in sample collection methods, the choice of percutaneous drainage instead of ERCP introduces variability that can distort microbiological results. Particularly, the use of an endoscopic sphincterotome during ERCP represents a risk of contamination from duodenal microbiota, potentially altering the true microbial composition of bile. Furthermore, the prophylactic administration of antibiotics prior to sample collection may compromise the native microbial population, limiting the accuracy of microbiota profiling. Moreover, various studies have employed heterogeneous analytical techniques: shotgun metagenomic sequencing, 16S rRNA gene profiling, and aerobic/anaerobic culture. This methodological variability limits the standardization, reproducibility, and comparability of results across different interventions. Conclusively, the paucity of data available regarding the physiological biliary microbiota in healthy individuals represents another limitation in determining the pathological shifts from normal microbial composition. The aim of future studies should be the standardization of sampling techniques and analytical approaches in order to obtain more robust evidence supporting the role of the biliary and gut microbiota as feasible diagnostic, prognostic, and therapeutic tools in hepato-biliary disease management.

## Figures and Tables

**Table 2:** Biliary and intratumoral microbiome findings in cholangiocarcinoma.

Reference (Year)	Study Population	Sampling Method and Sequencing Approach	Principal Taxa Increased in CCA (vs. Benign Disease)	Reported Diagnostic or Mechanistic Significance
Ito et al., 2020 [115]	41 malignant biliary strictures (mixed iCCA + pCCA + dCCA) vs. 33 benign strictures	ERC-aspirated bile; 16S rRNA metagenomics	Enterobacteriaceae family; colibactin-positive *E. coli*	DNA damage-mediated carcinogenesis; Enterobacteriaceae abundance as malignant biomarker
Chen et al., 2021 [7]	30 distal CCA vs. 30 choledocholithiasis	Intraoperative bile; 16S rRNA NGS	Enterobacteriaceae, *Staphylococcus*, *Klebsiella*, *Faecalibacterium*, *Corynebacterium*	Enterobacteriaceae enrichment plus higher α-diversity differentiate dCCA from benign obstructive disease
Di Carlo et al., 2022 [117]	50 extra-pancreatic biliary cancers (34 CCA, 16 ampullary) vs. 58 pancreatic cancers	ERC bile; 16S rRNA NGS	*Alcaligenes faecalis *↑, *Acinetobacter* spp. ↓ in CCA	Microbial signature improves discrimination between CCA and pancreatic ductal adenocarcinoma
Li et al., 2023 [118]	47 perihilar CCA, 29 distal CCA, 38 pancreatic cancer	ERC bile; 16S rRNA NGS	pCCA: *Pseudomonas*, *Sphingomonas*, *Halomonas* dCCA: *Streptococcus*, *Prevotella*, *Halomonas*	Distinct taxa panels permit molecular subclassification of malignant biliary strictures
Avilés-Jiménez et al., 2023 [119]	100 CCA (mixed sites) vs. 100 benign biliary disease	ERC brushings; 16S rRNA + qPCR for *Helicobacter*	*Helicobacter*, *Campylobacter*, *Prevotella*, *Fusobacterium*, Methylophilaceae	Chronic *H. pylori *colonization with virulence factors may drive progression from benign inflammation to CCA
Sitthirak et al. [120]	62 CCA tumor specimens pre-chemotherapy	Shotgun metagenomics of tumor tissue	Gammaproteobacteria over-represented in gemcitabine/cisplatin-refractory tumors	Bacterial metabolic inactivation of nucleoside analogs underlies primary chemoresistance

## Data Availability

No new data were created or analyzed in this study. Data sharing is not applicable to this article.

## Abbreviations

The following abbreviations are used in this manuscript:

AIH	Autoimmune Hepatitis
BA	Bile Acid
BSH	Bile Salt Hydrolase
CCA	Cholangiocarcinoma
CDT	Cytolethal Distending Toxin
dCCA	Distal Cholangiocarcinoma
dMBO	Distal Malignant Biliary Obstruction
HCC	Hepatocellular Carcinoma
iCCA	Intrahepatic Cholangiocarcinoma
NGS	Next-Generation Sequencing
PSC	Primary Sclerosing Cholangitis
PBC	Primary Biliary Cholangitis
SCFA	Short-Chain Fatty Acid
TGR5	Takeda G-Protein-Coupled Receptor 5
TLCA	Taurolithocholic Acid
UDCA	Ursodeoxycholic Acid
